# Conservation impacts and hidden actions in a randomized controlled trial of a marine pay-to-release program

**DOI:** 10.1126/sciadv.adr1000

**Published:** 2025-04-23

**Authors:** Hollie Booth, Thomas Pienkowski, M Said Ramdlan, Kusuma Banda Naira, Muhsin, E. J. Milner-Gulland, Luky Adrianto, Paul J. Ferraro

**Affiliations:** ^1^ Department of Biology, University of Oxford, Oxford, UK.; ^2^ The Biodiversity Consultancy, Cambridge, UK.; ^3^ Centre for Environmental Policy, Imperial College London, London, UK.; ^4^ Durrell Institute of Conservation and Ecology, University of Kent, Canterbury, UK.; ^5^ Department of Aquatic Resources Management/Center for Coastal and Marine Resources Studies, IPB University, Bogor, Indonesia.; ^6^ Yayasan Kebersamaan Untuk Lautan, Bali, Indonesia.; ^7^ Ministry of Marine Affairs and Fisheries, Jakarta, Indonesia.; ^8^ Johns Hopkins University, Baltimore, MD, USA.

## Abstract

Incentive payments could cost-effectively and equitably achieve biodiversity conservation goals but could also trigger unintended countervailing actions. Here, we report on a preregistered, randomized controlled trial of a pay-to-release program among small-scale, Indonesian fishing vessels for the release of two critically endangered marine taxa from fishing gear: hammerhead sharks and wedgefish. A conventional monitoring approach, which quantifies impacts based on conservation-relevant actions (i.e., numbers of live releases), implies that the program was successful: a 71 and 4% reduction in wedgefish and hammerhead shark mortality, respectively. The experimental data, however, imply that the pay-to-release program also induced some vessels to increase their catch, thereby decreasing wedgefish mortality by only 25% [confidence interval (CI): −49 to 10%] and increasing hammerhead mortality by 44% (CI: 8 to 92%). Our results do not imply that pay-to-release programs cannot work but rather demonstrate the complexity of designing incentive-based conservation programs and the importance of piloting them using experimental designs before scaling up.

## Teaser

Pay-to-release program encouraged live release of endangered marine taxa but led some fishers to increase catches of those taxa.

## INTRODUCTION

Of the 1 million species threatened with extinction, large, long-lived marine animals (“marine megafauna”) are among the most threatened and undermanaged. One group of marine megafauna—sharks, rays, and their cartilaginous relatives (class Chondrichthyes)—is among the most threatened vertebrate taxa on Earth, primarily due to overfishing in both targeted and bycatch fisheries (*1*–*3*).

Marine megafauna populations continue to decline (*1*–*3*) despite well-documented technologies and practices that reduce the impacts of fisheries on these populations [e.g., changes in fishing gear, technology, timing, and location, or through safe postcapture release (*4*, *5*)]. One reason why these technologies and practices are not widely adopted is socioeconomic barriers. For example, there can be direct costs or opportunity costs associated with mitigating catches or sociopsychological barriers to behavioral change, such as negative attitudes and social norms, cognitive biases, or a lack of perceived control in adopting (by)catch-relevant behavior (*6*, *7*). These barriers are particularly acute in small-scale mixed species fisheries (SSFs) in the Global South, where vessels commonly use unselective fishing gear in fishing grounds that overlap with ranges of endangered species, which means that vessels often catch endangered species even if they are not the primary targets (*6*, *8*). SSF households typically rely on marine resources for their livelihoods and assign consumptive use value to all catches—including endangered species (e.g., for food or income) (*7*–*10*). Households thus face trade-offs between conservation and livelihood objectives, where the need for food and income is at odds with mitigating catches of endangered species (*7*, *9*, *11*, *12*). In parallel, wealthier and more powerful ocean stakeholders (e.g., tourism industry and commercial fisheries) both benefit from and harm marine megafauna populations yet rarely contribute toward the costs of conservation (*13*–*15*). Overcoming these socioeconomic barriers and distributive issues is critical for achieving the Global Biodiversity Framework’s vision of “living in harmony with nature” and necessitates equitable conservation interventions, which fairly distribute the costs and benefits of conservation and deliver positive outcomes for biodiversity and people (*16*, *17*). A promising but untested way of achieving this goal is to use incentive-based approaches—such as compensatory payments for reducing fishing effort or rewards for live releases—targeted at SSFs and funded through levies on wealthier actors (*9*, *14*, *18*–*20*).

Conservation scientists and economists have long asserted, based on economic principles, that paying resource users to engage in conservation-relevant actions (whether through enhancing biodiversity or refraining from damaging it) is an efficient way to yield conservation benefits while also improving economic welfare (*9*, *19*, *21*–*23*). In a marine conservation context, this has been put into practice through pay-to-release schemes for threatened bycatch species in Kenya, India, and Brazil (*19*, *24*), with thousands of individuals reportedly released. Yet, the expected conservation benefits from incentive payments may not be unambiguously positive because of four countervailing pathways that can change behavior in unforeseen ways. First, incentive programs are plagued by hidden information, aka adverse selection (*25*). When some resource users already engage in the targeted conservation action in the absence of payments (e.g., not deforesting or not fishing) and conservation practitioners cannot identify those users in advance, payments can end up going to users who would have done the actions without payment (i.e., a lack of additionality). Second, incentive programs suffer from hidden actions, aka moral hazard (*25*), when users change their behaviors in ways that are unobservable to practitioners with the intent of securing more payments at the expense of conservation goals [e.g., wildlife compensation payments can encourage herders to deliberately expose their livestock to more predation risk (*26*)]. Third, when resource users are budget constrained, conservation payments can relax these constraints and enable greater levels of exploitation through the purchase of additional or more efficient equipment (e.g., more fishing nets and fuel) (*27*, *28*). Last, payments can induce spillovers to nontarget ecosystems or species. In the fisheries context, this problem is known as the “multiple margins” problem (*29*). For example, encouraging fishers to change gear to protect species A can have deleterious effects on species B (*30*, *31*). Moreover, these four pathways can interact and amplify each other. As such, incentive programs for threatened marine species need to be empirically evaluated rather than promoted without unambiguous evidence of their effectiveness (*32*).

However, quantifying the impacts of conservation incentives has been hindered because detecting these four countervailing pathways requires the estimation of counterfactual behaviors: How would the resource users have behaved in the absence of incentives? If the incentives are randomized [e.g., (*33*)], the control group’s behaviors provide an estimate of recipients’ counterfactual behaviors. Yet, incentive programs, like most environmental programs, are rarely randomized (*32*). In conservation, two notable exceptions took place in terrestrial ecosystems and aimed to reduce deforestation and improve land use practices (*33*, *34*). We know of no randomized controlled trial (RCT) of a species-based conservation intervention, and in the marine context, we know of no RCT for any conservation intervention, much less an incentive-based program.

Here, we report on an RCT of a marine conservation program, which used an incentive-based intervention to deliver positive outcomes for biodiversity while ensuring that participants were no worse off.

### Study design

**Fig. 1.**
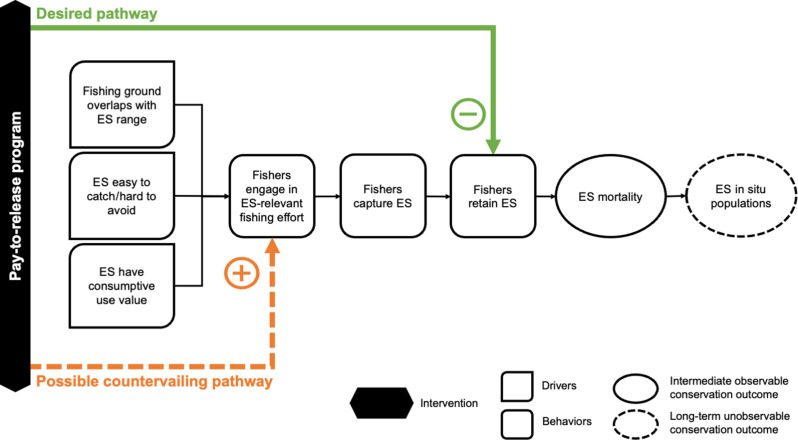
Conceptual diagram of fisher behavior. A simplified theory of change describing the local-level drivers of fisher behaviors leading to endangered shark species (ES) mortality in SSFs and the desired and potential undesired pathways under a pay-to-release program, which aims to incentivize fishers to reduce their retention of ES.

Theory and empirical evidence suggest that a pay-to-release approach could cost-effectively achieve marine megafauna conservation goals while ensuring that coastal communities are no worse off (*9*, *19*, *24*). Pay-to-release not only compensates fishers for the economic opportunity costs of marine conservation (*9*, *11*) but also addresses a key challenge in designing payments for environmental service programs: ensuring that the incentives are individually targeted, timely, and conditional while also ensuring that there is a clear mechanistic link between the incentivized behavior and the desired population-level conservation outcomes, which can be difficult to observe, take time to accrue, and can be influenced by external factors (*21*, *35*). However, this mechanistic link can be countervailed by hidden actions, multiple margins, and relaxation of budget constraints (Fig. 1) [hidden information is not a threat in our study context because vessels do not release the target taxa alive in the absence of incentives; catching and domestically trading and consuming the taxa remain legal, lucrative, and socially legitimate (*7*, *9*)]. This ambiguity warrants experimental evaluation of pay-to-release programs (*32*).

We evaluate a program that incentivized live releases for two critically endangered taxa, hammerhead sharks and wedgefish, in five villages across two Regencies in Indonesia: East Lombok and Aceh Jaya. The taxa and country are global conservation priorities for which fisheries interventions are urgently needed because overfishing is the greatest threat to hammerhead sharks and wedgefish, and Indonesia catches more sharks and rays than any other nation (*2*). Moreover, although international trade in hammerhead sharks and wedgefish is regulated under the Convention on International Trade of Endangered Species, there are no laws that regulate the capture and domestic use of these taxa in Indonesia. Therefore, domestic catches of these endangered taxa continue unabated throughout Indonesia (*36*). The study locations are known hotspots for hammerheads and wedgefish catches, wherein fishers use two of the highest-risk gear types for the capture of sharks and rays globally: gill nets and longlines (*3*, *37*). These fisheries are representative of a large proportion of the fisheries in Indonesia (and globally), where >90% of Indonesia’s capture fisheries fleet is small scale, and in this fleet, most use unselective nets and lines (*38*). The study taxa also play important roles in the livelihood strategies of local fishers in the study villages, making a pay-to-release intervention suitable for alleviating socioeconomic barriers to conservation-relevant behavior (*7*, *9*, *11*). This context is also representative of many of the world’s largest shark-fishing nations in the Global South, such as India, Malaysia, Sri Lanka, and Bangladesh (*39*–*42*). SSFs are ubiquitous throughout the coastal waters of these nations, and similar regulatory gaps and socioeconomic barriers persist, such that critically endangered sharks and rays are frequently caught and act as important sources of food and income for coastal communities (*39*–*42*).

The pay-to-release program was designed using an evidence-based and participatory process, where preintervention research conducted within the target villages indicated that pay-to-release was a feasible and cost-effective approach for delivering conservation and well-being outcomes (*7*, *9*). Before implementing the program, vessels did not release live hammerheads or wedgefish, and no other endangered species conservation programs were being implemented for our target taxa during the study period. In the program, vessels could receive payments conditional on the submission of live release videos (e.g., movie S1) taken on program-provided cameras (one camera per vessel). To ensure that the released fish were alive and the videos had not been duplicated or tampered with, the program staff checked and verified the videos, including the metadata (i.e., time and date of recording).

On the basis of prior research, fishers in Aceh Jaya were offered IDR 15,000 (small fish) or 50,000 (large fish) per hammerhead (~$1 or $3.25, respectively) and a fixed rate of IDR 120,000 per wedgefish (~$8). Fishers in East Lombok were offered IDR 500,000 per hammerhead and IDR 2,000,000 per wedgefish (~$33.50 and $134, respectively) (*9*, *11*). These differentials were based on information about how much vessels could receive in local markets (*9*, *11*).

The program conducted a Free, Prior, and Informed Consent (FPIC) process to recruit fishers to participate, and the RCT was preregistered. The program was implemented with 87 vessels (East Lombok, *N* = 50; Aceh Jaya, *N* = 37) over a 16-month time period (May 2022 to August 2023) using a two-sequence, four-period, two-treatment crossover design, with fishers blocked by village and randomized into two sequences (see Materials and Methods) (*43*).

To measure conservation-relevant actions, we used the count of live releases from the videos that fishers submitted to receive payments (e.g., movie S1). To measure conservation outcomes, we used retained catch (a proxy for mortality) of hammerheads and wedgefish, observed via daily landings surveys for all 87 vessels in each village throughout the entire study period. To contextualize the quantitative data, we also conducted semistructured interviews with fisher households before, during, and after the intervention (see Materials and Methods). We recorded project costs following Iacona *et al.* (table S1) (*44*).

## RESULTS

### Conventional monitoring and evaluation

Evaluations in conservation programs conventionally use nonexperimental approaches that rely only on data under the treated (i.e., payment) condition. This approach would assume that fishing behaviors with and without live release payments are the same except for the live releases induced by the payments. In other words, the approach assumes that there are no countervailing pathways (Fig. 1), and thus the live releases represent the conservation impact of the program.

**Fig. 2.**
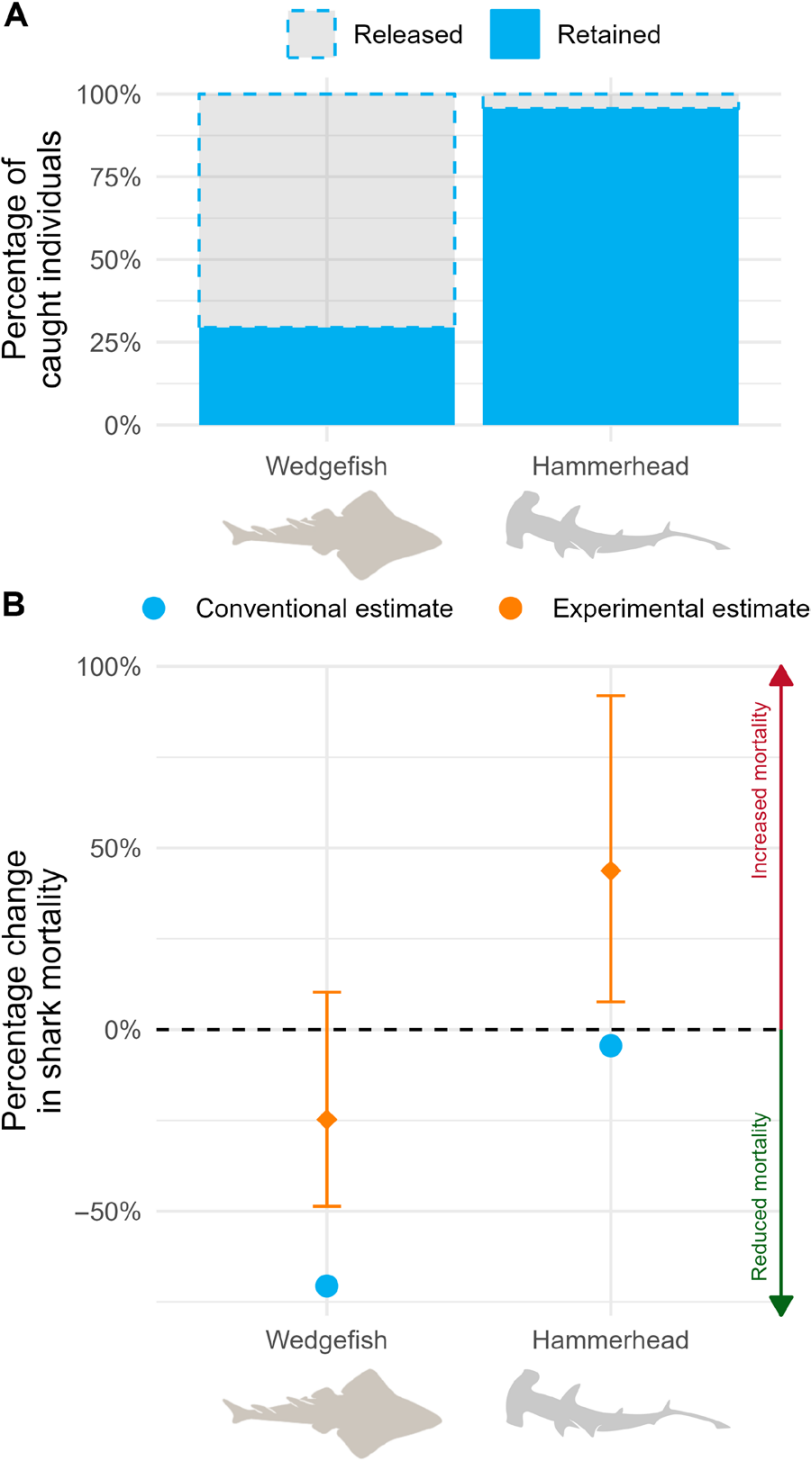
Estimated conservation impacts of the pay-to-release program on wedgefish and hammerhead mortality under conventional and experimental monitoring and evaluation approaches. (**A**) Estimated impacts on fish mortality under the conventional approach, which relies on only the observations of live releases and assumes no countervailing behavior on fishing effort triggered by the offer of payments. (**B**) Estimated impacts on fish mortality under the experimental approach, which quantifies the difference between the retained catch (mortality) under the treatment and control conditions and reflects the combined effects of live releases and countervailing behaviors.

This approach yields an estimate of an average treatment effect (ATE_C_, where C denotes “conventional” monitoring) for each taxon by dividing the number of released fish by the total number of fish caught (Eq. 1 and Materials and Methods). With 475 wedgefish released (i.e., alive) and 198 retained (i.e., dead), the ATE_C_ for wedgefish is a 71% reduction in mortality. With 364 hammerheads released and 7830 retained, the ATE_C_ for hammerheads is a 4% reduction in mortality (Fig. 2A).

However, these estimated ATE_C_ values represent only a partial picture of the true impact of the program if the payments affected fishing behaviors beyond just the live releases [i.e., if no countervailing behavioral pathways (Fig. 1) were triggered, the ATE_C_ represents the maximum reduction in mortality]. Any countervailing behavioral pathways that induce vessels to catch more of the targeted taxa would shift the net program impact toward zero or even into a negative conservation impact (i.e., an increase in net mortality).

### Experimental monitoring and evaluation

To estimate average treatment effects that include the effects of countervailing behavioral pathways, we used the full experimental dataset of vessels under treated and control conditions (ATE_E_, where E denotes “experimental” monitoring). In the experiment, the control condition provides an estimate of the counterfactual vessel behaviors in the absence of the payment program. By comparing ATE_E_ with ATE_C_, we can directly test the assumption that payments induced no changes in fishing behaviors except for the live releases. Our null hypothesis, which assumes no countervailing behavioral pathways (i.e., the payments only incentivized live releases), is that ATE_C_ is equal to ATE_E_. If, however, ATE_E_ were significantly smaller in magnitude than ATE_C_ (i.e., a lower reduction in retained catch), we would infer the presence of countervailing behaviors that attenuate the net program impact.

Using the full experimental sample, we conclude that ATE_E_ < ATE_C_, and thus countervailing behaviors are present. In Fig. 2 (bottom panel), we present the estimated ATE_E_ (orange) and its 95% confidence interval (CI) for each taxon and compare it to the ATE_C_ (blue). The results show that the pay-to-release program decreased retained wedgefish by an estimated 25% (CI: −49 to 10%) and increased retained hammerhead catch by an estimated 44% (CI: 8 to 92%) (i.e., an increase in mortality; a negative conservation outcome). Using chi-square tests, we can reject the null hypothesis that ATE_C_ = ATE_E_ for hammerheads (*P* < 0.01) and for wedgefish (*P* < 0.01).

Although the estimated CI for wedgefish does not exclude a conservation-relevant reduction in mortality, these results nonetheless imply that one or more of the countervailing behavioral pathways in Fig. 1 are active in the pay-to-release program: The offer of payments induced fishers, on average, to increase their fishing effort in ways that led to more at-vessel catch of the target taxa than would have happened in the absence of payments. Consistent with this conclusion, we see these patterns only for the subsample of vessels that received at least one payment (i.e., vessels that released live fish) (tables S2 and S3). We also draw the same conclusions after removing potential outlier observations and after using alternative statistical estimators of ATE_E_ (tables S2 to S4).

### Interrogation of causal assumptions

These inferences are based on several causal assumptions (*45*), which are summarized in Materials and Methods (Table 1). Here, we assess the plausibility of the two most important assumptions: excludability (i.e., the process by which treatments are assigned has no effect on potential retained catch except through its effect on a vessel’s treatment status) and no interference (the retained catch of a vessel depends only on its own treatment status but not on the treatment status of other vessels).

**Table 1.** Causal assumptions required for making causal claims from our study’s RCT. N.A., not applicable.

Assumption	Potential violations in study design	Features of design or analysis that address potential violations
Excludability: The process by which treatment is assigned has no effect on potential outcomes except through its effect on a unit’s treatment status	Trial engagement effects	Payments were not conditional on retained catch. Monitoring of retained catch was by trusted team and identical under treated and control conditions.
	Nonrandom missing landings observations	No evidence of nonrandom missing landings observations (supplementary text 1.2).
No interference: The potential outcome of a unit depends only on its own treatment status but not on the treatment status of another unit	Within-vessel interference in the form of “carryover” effects in the crossover design.	Clear start and stop dates for experimental conditions.
		Analysis using only first-period data.
	Across-vessel interference in the form of price-mediated effects, stock-mediated effects, or location-mediated effects.	Field knowledge of local markets, fisheries, and vessel choices.
		Analysis robust to some forms of across-vessel interference.
No multiple versions of treatments: The treatment must be consistent among all treated units	Although the payment program structure was identical for all vessels, the payment amounts varied by Regency, according to variation in opportunity costs of live releases.	Treatment was defined as an offer of a live-release payment at an amount appropriate for the Regency, and thus, multiple versions are not an issue in the analysis.
		In the exploratory analysis, we estimated the impact by Regency.
No noncompliance: Units receive the treatment to which they were assigned	N.A. The treatment was an offer of a live-release payment. The field team ensured fidelity to treatment assignment across periods.	N.A.

The most plausible violation of the excludability assumption would be a trial engagement effect, whereby vessels hide their retained catch from the monitoring team under the treated condition but not under the control condition [e.g., an observability or social desirability bias (*46*), whereby fishers, believing that the measurement team perceived retained catch as undesirable, hid retained catch only under the treated condition—if hiding occurred under both conditions, this behavior would threaten the external validity of the design but not its internal validity].

This form of trial engagement effect is unlikely in our context for three reasons. First, the incentive payments were not conditional on catches, so fishers under the treatment condition were not incentivized to hide their catches. Second, the behavior was not illegal or sensitive. Third, the retained catches were monitored by people who were well known to the communities and had been collecting fisheries data for several years prior to the pay-to-release program. Moreover, the higher retained catch of hammerheads under the treated condition is empirically inconsistent with fishers hiding retained catch. The only way to be sure that such a violation does not exist in a crossover design would be to use remote at-vessel monitoring (*47*), which would likely be prohibitively expensive to implement across many small-scale vessels and comes with its own risks regarding community relations and research ethics (*48*). Another plausible violation of excludability is if treatment status was correlated with missing landings observations, but we find no evidence in the patterns of missingness between treated and control conditions (supplementary text 1.2).

There are two forms of interference that could have occurred in our design. First, in a crossover design, there could be within-vessel, across-periods interference (aka carryover effects, whereby a vessel’s treatment condition in one period affects its own behavior under the control condition, or vice versa). To mitigate this threat in the design, the field team clearly and repeatedly communicated to the vessels the start and stop dates for the experimental conditions. In the analysis, we can reestimate the program impact using only the Period 1 data and eliminate the threat that treatment exposure in one period could carry over into subsequent treatment or control conditions. We draw the same conclusions (tables S2 and S3). We cannot, however, use our data to eliminate a form of within-vessel interference in which a vessel’s expectation of future treatment exposure affects its behavior under the control condition. The most plausible mechanism for such an effect would require a well-functioning credit market, whereby control vessel owners could borrow funds against their expected future release payments and then invest those funds in a way that would affect the retained catch of the two target taxa. Such a mechanism is unlikely to be operative in our study context (supplementary text 1.3).

The second possible form of interference is across-vessel interference, where a vessel’s behaviors under treated or control conditions are affected by another vessel’s treatment status. This interference could occur through market-mediated behavior changes (e.g., the treatment induces a change in the supply of certain species, which subsequently affects prices), stock-mediated changes (e.g., the treatment changes the stock of fish that are available for other vessels to catch), or location-mediated behavior changes (e.g., treatment induces a change in where vessels fish, which subsequently affects the retained catch of other vessels via displacement of vessels to other areas). Although we cannot rule out these forms of interference with certainty, field knowledge indicates that they are unlikely. Any stock-mediated or location-mediated effects are likely to be small because the life history traits of wedgefish and hammerheads are slow, and fishing is spread out over large spatial scales. Price-mediated effects are also unlikely because the markets are not strongly competitive, buyers are not responsive to short-term supply changes, and vessels are not responsive to short-term changes in price (*11*). Nevertheless, we note that the estimated ATE_E_ changes little if we use an alternative statistical estimator that eliminates price-mediated effects under the assumption that these effects are constant across periods because the proportion of treated and control vessels across periods is constant (see supplementary text 1.3 for more details on all of these potential forms of interference).

### Exploratory analyses of mediating pathways

The RCT was not designed to estimate mediating effects, but we use exploratory analyses to shed light on the potential pathways that attenuated the program’s conservation outcomes despite its positive effect on live release behavior (supplementary text 1.4).

The exploratory analysis suggests that the countervailing pathways, at least in part, were a mix of hidden actions induced by the wedgefish payments (i.e., increases in wedgefish-relevant fishing effort) and a subsequent deleterious spillover to the hammerhead catch. In the analysis, we observe that, on average and within each vessel, retained hammerheads and wedgefish are positively correlated (ρ = 0.44) in the subset of the three villages in Aceh Jaya that received most of the payments (~99% of payments), and the correlation is 37% higher under the treatment condition than under the control condition. These patterns, however, are not seen in the East Lombok villages (around ρ = 0.05 under both conditions), where very few releases were reported. Moreover, we detect an increase in hammerhead mortality among the villages that received most of the wedgefish payments, although vessels in these villages rarely released live hammerheads. As we describe in Discussion, a mix of hidden actions induced by the wedgefish payments and a subsequent spillover to the hammerhead catch is plausible, given that wedgefish payments were larger than the hammerhead payments, and additional wedgefish catches can be more easily mitigated through live releases due to their relatively high postcapture survivability, whereas hammerheads exhibit lower survivability (*9*, *49*, *50*). The patterns in the data are not consistent with a broad increase in fishing effort induced by a relaxation of vessel budgets. Interviews with fishers and their families provided qualitative evidence consistent with these conclusions (supplementary text 1.5).

## DISCUSSION

The results from an RCT of a pay-to-release program suggest that, although the payments did induce conservation-relevant actions, they also induced countervailing pathways within some vessels, which attenuated the conservation benefits from these actions. These findings illustrate common challenges and trade-offs in conservation incentive schemes (*21*, *25*). Research in the program design phase sought to estimate optimal payment rates that would incentivize behavior change and ensure that fishers were no worse off (*9*), but the payment rates may have been too low for fishers in East Lombok, where very few releases were reported, and too high in Aceh Jaya, where payments triggered perverse countervailing behaviors among some vessels.

The observed heterogeneity across taxa and study sites is likely explained by a combination of ecological, technical, and socioeconomic factors. In Aceh Jaya, where net-using vessels catch a range of fish sizes, the market values of sharks and rays typically vary by size. In contrast, the guaranteed live release payment was based on an average value across size classes, which could have incentivized the intentional catch and release of smaller individuals with low market value. Consistent with this hypothesis, smaller individuals appeared to make up most of the live releases based on the release videos. Because wedgefish and hammerheads co-occur in Aceh Jaya waters, any incentive to catch more of one taxon leads to increased catch of the other. For wedgefish, additional catches can be more easily mitigated through live releases because their buccal pump respiratory system confers relatively high postcapture survivability, whereas hammerheads exhibit lower postcapture survivability because their ram ventilation respiratory system requires them to swim constantly to avoid suffocation (*9*, *49*, *50*). Thus, the additional hammerhead catches could not be released alive, leading to an overall increase in hammerhead mortality. In contrast, live releases were rare in East Lombok. Interviews with fishers suggest that live releases were hampered by technical and economic constraints. Although participants generally reported positive attitudes toward the intervention (supplementary text 1.5), they reported that the target taxa were typically dead once removed from their longline gear or that the compensation was not high enough to match the market prices in their Regency.

Our results do not imply that a pay-to-release program cannot work in Indonesia, but rather they offer valuable insights into how such programs could be more effectively designed and deployed. Potential changes to the program in Aceh Jaya include capping the number of compensated releases per week (e.g., based on typical catch rates observed under the control condition) and differentiating payments by size classes. Another option could be to supplement the individually targeted, action-oriented incentives with community-level, outcome-oriented incentives. For example, eDNA or Baited Remote Underwater Videos could be applied to inexpensively monitor relative abundance of sharks and rays (*3*, *51*) to which additional community-wide rewards could be tied to incentivize outcomes as well as actions. In East Lombok, fishers could be offered higher compensatory payments to explore whether behavioral changes are triggered, or it may be the case that live release is not a sufficiently effective mitigation measure for longlines due to high postcapture mortality. Pay-to-release generally appears to be most viable for wedgefish captured in gill nets, which is also consistent with previous biological studies on shark respiratory physiology and survivability and fisher perceptions of survivability (*9*, *49*, *50*). For hammerheads, other measures to avoid and minimize capture may be critical, such as spatiotemporal closures and gear swaps. Future studies could experimentally compare the effectiveness of different interventions and incentive structures if these variations were implemented using randomization. Program design could also be strengthened through more in-depth research to understand and quantify causal mechanisms. This could be supported through, for example, analyzing demographic and sociopsychological predictors of observed behavior [e.g., (*52*, *53*)]. Additional data collection on trends in market prices, fishing grounds, at-sea fishing behavior, and relative abundance of fish in the study and nonstudy villages could also help to better understand and quantify hidden actions, as well as provide wider contextual information to assess the plausibility of key causal assumptions in RCTs. Last, a longitudinal study, in which individual vessels experience multiple control and treatment periods over time, could be used to test for interactions between period and treatment and explore potential learning effects.

More broadly, even if pay-to-release programs were designed to be more effective, they could also be complemented by other interventions and policy tools. For example, it may be that measures to avoid and minimize the capture of endangered species—such as spatiotemporal closures and gear swaps—would be a first choice where feasible (especially for hammerheads), with live release being a last resort in a mitigation hierarchy of actions (*4*). Complementary policy tools, such as regulatory changes, individual transferable quotas, and bycatch levies in the commercial sector, could support the wider enabling environment for bycatch mitigation and provide monetary incentives for community-based conservation while holding wealthier and more powerful ocean stakeholders accountable (*14*, *54*).

Given the importance of program design and implementation context, we do not wish to speculate about the degree to which our results generalize to other areas or time periods. Our RCT, in only five villages and two Regencies, is the first and only RCT of a pay-to-release program (and on any kind of incentive program in the marine context). To strengthen its external validity, more RCTs of marine conservation interventions are needed, as is a better understanding of the contexts in which these interventions may be most appropriate and effective and which factors facilitate or inhibit successful outcomes and wider scaling.

Our RCT does, however, offer an important generalizable lesson on the importance of piloting conservation programs with experimental designs before scaling the programs up, as well as the importance of creating systems that facilitate learning and adaptive management (*32*, *55*). Even the most careful efforts to design a conservation program may not account for all types of real-world uncertainties and complexities. For example, although predictive, participatory research based on surveys and focus groups (*9*) is an important first step for designing conservation programs (especially for gaining trust, buy-in, procedural justice, and consent), it also has limitations. In our preintervention research stage, almost all fishers said that they would release live fish at the program’s payment levels, yet many did not (i.e., the previous studies were prone to hypothetical bias). Moreover, in the same preintervention research stage, fishers implied that wedgefish and hammerhead catches were stochastic and could not be avoided with changes in fishing behavior (*9*). The RCT results, however, imply that fishing practices could be changed to affect catches, and thus, some specialist information was withheld by fishers during the surveys (e.g., information on operational or tactical factors that influence catch). When theory and field experience cannot unambiguously predict outcomes, piloting conservation programs with experimental designs is particularly important (*32*, *56*). If the pay-to-release program staff had relied solely on monitoring conservation actions without an experimental estimate of counterfactual behaviors [as is typically the case in conservation programs (*32*, *57*), including current examples of pay-to-release programs (*19*, *24*)], the staff would have concluded that the program was successful. Moreover, given hundreds of live releases and the fact that many vessels released no live fish, the next steps would likely have focused on scaling up the program and finding ways to increase vessel participation. Using the experimental design, however, allowed the staff to identify perverse incentives, which would have otherwise gone undetected and which countervailed the conservation benefits from live releases. Rather than implying that the program and participation should be scaled up, those results instead implied that the program should be redesigned. Piloting programs with experimental designs can thus help identify real-world pitfalls and refine interventions before they are scaled up.

Although many conservation practitioners shy away from experimental designs for practical and ethical reasons and possibly because demonstrable failure may lead to the loss of donor support, our study highlights the importance of building in robust impact evaluation from the outset of new interventions, preregistering trials, and reporting failure and learning from it (*32*, *58*, *59*). Our experimental design also offers a pragmatic compromise between scientific and ethical obligations that could be adopted by others. Wider adoption of pragmatic experimentation can establish a culture of continuous learning that can create a more robust body of empirical evidence to guide practitioners on where to spend limited conservation funding. This evidence will ensure that conservation interventions can better contribute to bending the curve on biodiversity loss and delivering the Global Biodiversity Framework’s 2050 vision of “living in harmony with nature.”

## MATERIALS AND METHODS

### Study sites

**Fig. 3.**
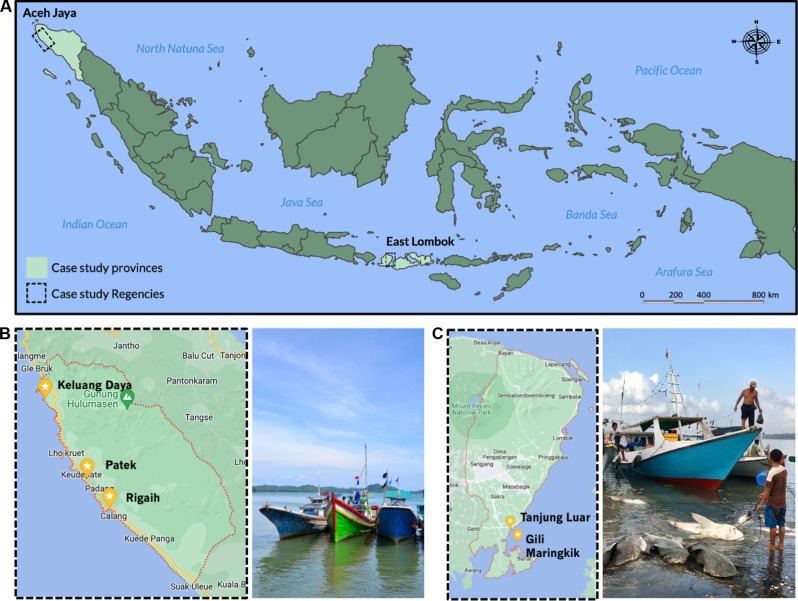
Maps of study sites. (**A**) Map of Indonesia, showing locations of study provinces/Regencies. (**B**) Map of Aceh Jaya Regency (left) indicating case study villages and an illustrative photograph of fishing boats in the Regency (right). (**C**) Map of East Lombok Regency (left) and an illustrative photo of fishing boats in the Regency (right). Credit: H.B., University of Oxford.

The study sites were five villages in two Regencies: Keluang Daya, Patek, and Rigaih in Aceh Jaya Regency (Aceh Province) and Tanjung Luar and Gili Maringkik in East Lombok Regency (West Nusa Tenggara Province; Fig. 3). The fisheries in these Regencies represented contrasting case types in terms of technical and ecological characteristics (i.e., gears used and fisheries ecology) and socioeconomic contexts (e.g., culture, income, and market access) (*7*, *9*, *11*, *60*). In Aceh Jaya, small vessels conduct short (1 to 3 days) trips in coastal waters, using bottom gill nets mainly to target reef and demersal fish. Juvenile hammerheads and wedgefish are incidentally caught and then sold and consumed as part of overall livelihood strategies. Hammerheads and wedgefish make up 10 to 30% of total catches (by volume) depending on the season although a relatively smaller proportion of the total catch value because most of this volume consists of low-value juvenile hammerheads (*7*). Hammerheads and wedgefish meat are typically consumed locally, whereas the fins of large wedgefish (which are rarely caught) are purchased by specialized collectors. In East Lombok, specialized shark longline vessels conduct 10- to 20-day fishing trips in pelagic waters, targeting large, mature sharks, and rays. The primary target taxa are large requiem (Carcharhinidae spp.) and mackerel (Lamnidae spp.), which make up >80% of total catch, whereas hammerheads and wedgefish consist of <10% of total catch (*11*). The catch is sold for domestic and international meat and fin markets. The fins are primarily for high-value export, whereas meat and other commodities (skin, cartilage, and liver oil) are consumed locally, domestically, or internationally (*7*, *9*, *11*, *60*).

### Study taxa

The pay-to-release program focused on incentivizing catch mitigation for two critically endangered taxa: hammerhead sharks (*Sphyrna* spp.) and wedgefish (*Rhynchobatus* spp.), which represent contrasting case types in terms of biological traits, fisheries ecology, and use values (*61*, *62*). Notably, both taxa have high catchability: wedgefish due to their relatively large total length and hammerheads due to their cephalic morphology, making them particularly vulnerable to capture in unselective gears such as gill nets. However, family Rhinidae exhibits high postcapture survivability of up to 90% depending on gear/conditions, whereas family Sphyrnidae exhibits low postcapture survivability of around 50% (*9*, *49*, *50*). The “white” fins of wedgefish are considered the best quality fins and are among the highest valued in the shark fin trade, whereas hammerheads are of moderate value (*11*).

### Program and study design

**Fig. 4.**
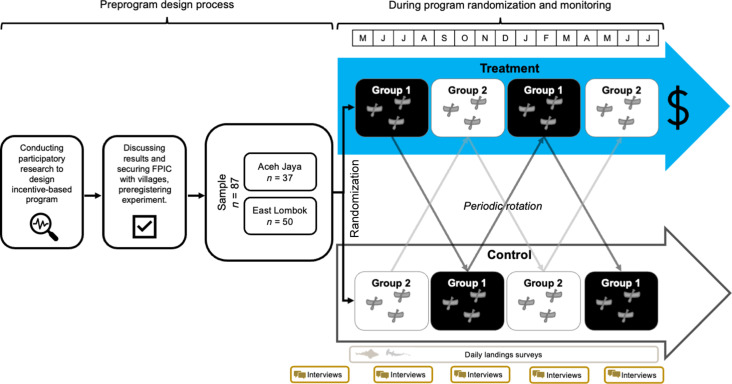
Conceptual diagram of our RCT experimental design.

An evidence-based and participatory process was used to design the program based on previous research (*7*, *9*) (Fig. 4). We conducted an FPIC process with target fishers (voluntary contracts available at https://osf.io/b27ja/?view_only=39fe7b0d223547a8a2cb92e217cdea99) and preregistered the RCT via AsPredicted (#93480, available at https://aspredicted.org/4T9_77M).

The RCT took place over a 16-month period (May 2022 to August 2023) and used a two-treatment, two-sequence, multiperiod, crossover design, with fishers blocked by village and randomized into two sequences (Fig. 4) (*43*). The field team started in May 2022 with 61 vessels in three villages and randomized vessels into one of two two-period sequences (TC or CT, where TC was the treated condition followed by the control condition, and CT was the opposite pattern). After randomization in May 2022, the program received more funding, and the field team was able to add 26 more vessels from two additional villages (Patek and Keluang Daya) and extend the duration of the experiment (number of periods). These 26 new vessels were randomized to their sequences after Period 1 ended for the first 60 vessels. In other words, the beginning of Period 2 for the original 60 vessels is the beginning of Period 1 for the added 26 vessels. The trial ended in August 2023, with the first 61 vessels exposed to a four-period sequence (TCTC or CTCT) and the other 26 vessels exposed to a three-period sequence (TCT or CTC). This sample of 87 vessels (East Lombok, *N* = 50; Aceh Jaya, *N* = 37) represented 100% coverage in these villages of vessels that used the highest risk gears for catching the study taxa.

The crossover design was chosen for statistical, ethical, and practical reasons (*58*). From a statistical perspective, it provided both within- and between-vessel variation in exposure to the release payments, which increased statistical power. From an ethical perspective, it ensured that all fishers had the opportunity to receive live release payments. From a practical perspective, the simple rotation design maximized simplicity and ensured that all fishers understood how the scheme would work. In Table 1, we summarize the relevant assumptions for making causal inferences from this experimental design (*45*), as well as how any potential violations are addressed in the design or the analysis.

### Data collection

Data were collected by trained local researchers with ethical approval by the Interdivisional Research Ethics Committee at the lead author’s institution (H.B.) (ref. R66416/RE001) and Indonesia’s National Research and Innovation Agency (BRIN) (refs. 407/E5/E5.4/SIP/2019, 39/SIP/IV/FR/1/2023, and 17/SIP.EXT/IV/FR/5/2023).

The biodiversity outcome for the analysis was retained catches of hammerheads and wedgefish at each vessel’s landing site (i.e., dead hammerheads and wedgefish on the vessel), which is a proxy for mortality and which was observed by the field team via daily landing site surveys for all 87 vessels in each village throughout the entire study period (fig. S1).

During a period, some vessels may never come to the landing site, and thus, their retained catch was not observed for that period. The field team observed the landing of 52 vessels in every one of the vessel’s experimental periods and the landing of 79 vessels in at least one treated and one control condition. One vessel was not observed at a landing site during the experiment, and thus, the sample size for the analysis is *N* = 86. On the basis of (i) field knowledge of why some vessels were not observed to land in some periods and (ii) the lack of a meaningful difference (statistically or numerically) between the numbers of observed periods and period lengths in treated and control conditions (supplementary text 1.2), we assume that the landings data were missing independent of potential outcomes. The unit of analysis was each unique vessel in each exposure period (“vessel-period”); total retained catch and live releases were calculated by aggregating observed retained catch and live release for each vessel within a given phase (fig. S2). Therefore, the sample for empirical analysis comprised 261 vessel-period observations representing 27,992 days of observations across all vessels by the field team at the landing sites (treatment = 14,269; control = 13,723).

### Estimation procedures

#### Nonexperimental conventional monitoring and evaluation approach

Using the live release data, we estimate the ATE_C_ for each taxon by dividing the number of live releases (*L*) by the total number of fish caught, which is the total of retained catch (*R*) plus live releases for each vessel (*i*) and period (*p*) (Eq. 1)

$${\text{ATE}}_{C}=\frac{\Sigma L_{\mathrm{ip}}}{\Sigma \left(R_{\mathrm{ip}}+L_{\mathrm{ip}}\right)}$$ (1)

#### Experimental evaluation

##### Estimand (target causal parameter)

For each taxon, we seek to estimate the average percent change in retained catch from offering live release payments or, equivalently, the average percent change in retained catch when vessels are switched from the control condition to the treated condition

$${\text{ATE}}_{E}=\frac{E\left[R{\left(1\right)}_{\mathrm{ip}}-R{\left(0\right)}_{\mathrm{ip}}\right]}{E\left[R{\left(0\right)}_{\mathrm{ip}}\right]}$$ (2)

where *R* is the retained catch, *R*(1) is the potential retained catch under treatment, *R*(0) is the potential retained catch under control, *E*[◦] is the expectation operator, *i* is the vessel, and *p* is the period. Given the field team randomized the offer of payments, we can use observable quantities of retained catch to estimate counterfactual quantities.

##### Statistical estimator

To estimate the ATE_E_, we use what some authors call a Poisson quasimaximum likelihood estimator (*63*). This Poisson estimator is appropriate for an outcome variable like retained catch (**r**), where the expected value of **r** conditional on covariates is nonnegative for all vessels. For the *i*th vessel from the *j*th village in the *p*th period, the conditional mean of this Poisson estimator is specified as

$$E\left[r_{\mathrm{ip}}|t_{\mathrm{ip}},v_{\mathrm{jp}},\beta ,\delta ,\alpha_{i}\right]=\alpha_{i}\text{exp}\left(\beta \;{\text{t}}_{\mathrm{ip}}+v_{\mathrm{jp}}^{\prime }\;\delta \right)$$ (3)

where **r** is the retained catch, **t** is a dummy variable for the treatment (equal to one if vessel was offered a pay-to-release payment in the period), β is the slope parameter of the treatment, **v** is vector of an interaction terms of dummy variables for each village (*j* = 1, 2, 3, 4, 5) and dummy variables for each period (*p* = 1, 2, 3, 4) (i.e., time-varying differences in retained catch across villages in each of the experimental time periods), δ is the slope parameter for the village-period interaction terms, **α** is an unobserved, vessel-level random effect (i.e., a time-constant, vessel-level error term that allows the constant in the model to vary by vessel), and exp is the exponential function. The vessel-level random effects and period-village interaction variables are included to increase the precision of the estimates. We assume that the random effects follow a gamma distribution (in analysis code available online, we confirm robustness of results under an alternative assumed Gaussian distribution). The village-period interaction variables also help in two other ways: They ensure that the standard error estimation reflects (i) the block randomization within villages and (ii) the variation in the season in which a period takes place in a village (for logistical reasons, not every village started and ended a period on the same date). We further adjust (weight) the estimation for minor (a day or two) differences in vessels’ period lengths within villages (i.e., exposure length). This estimator also uses cluster-robust estimated standard errors (clustered by vessel), which allows for arbitrary conditional variance and serial correlation (*63*). In other words, the estimator is robust to both serial correlation among the within-vessel observations (i.e., the errors in retained catch are serially correlated across time within a vessel) and to distributional misspecification (e.g., heteroskedastic errors and overdispersion, which violates the assumption in traditional Poisson model variance estimators that the conditional mean of **r** equals the conditional variance of **r**).

Given that treatment (**t**) is randomized, and thus the errors of the estimation model are uncorrelated with treatment status, our estimator provides an unbiased estimator of the ATE_E_, which is obtained from the model output by exponentiating the coefficient on the treatment variable (β) and subtracting 1 (also known as the incidence rate ratio). The exploratory analyses use the same estimator (supplementary text 1.4). To implement the estimation procedure, we use Stata v.18. See online analysis code for more details about this estimator and estimators used for robustness checks.

##### Robustness checks

To assess the robustness of our estimated ATE_E_, we conduct six additional analyses (see also supplementary text 1.1).

1) Period 1–only Analysis (col 2, tables S2 and S3): To assess the potential influence of carryover effects, we recalculate the ATE_C_ using only the Period 1 data and reestimate the ATE_E_ using the same data (recognizing that statistical power will be much lower with fewer observations and without the within-vessel variation under treatment conditions).

2) Recipient-only Analysis (col 3, tables S2 and S3): To confirm our expectations that the patterns that we observe in the full dataset would be stronger among the subgroup of vessels that engaged in live releases, we recalculate the ATE_C_ using only vessels that released live fish and reestimate the ATE_E_ using the same data (recognizing that statistical power declines with fewer observations).

3) Alternative ATE Estimator 1 (col 4, tables S2 and S3): Some conservation scientists are more familiar with a multilevel, mixed-effects estimator that assumes that the random effects follow a Gaussian (normal) distribution. To show that the results from this estimator are nearly identical to those in Fig. 2, we reestimate the ATE_E_ with this estimator and cluster-robust standard errors.

4) Alternative ATE Estimator 2 (table S4): To show that our conclusions are not an artifact of the Poisson estimator, we reestimate the ATE_E_ with a random-effects generalized least-squares (GLS) panel data estimator, which is unbiased given the randomization of the treatment (i.e., strict exogeneity is satisfied) but less efficient than the Poisson estimator (and it will not predict retained catch as well).

5) Alternative Variance Estimator (col 5, tables S2 and S3): To show that our inferences with the Poisson quasimaximum likelihood estimator are robust to violations of the assumptions that are required for the cluster-robust estimator, we estimate cluster-robust bootstrapped standard errors, which puts even less structure on the variance-covariance matrix values.

6) Influential Observations (col 6, tables S2 and S3): To show that our conclusions are not an artifact of a few influential observations in the tail of the retained catch distribution, we drop the top 1% of retained catch counts and reestimate the ATE_E_. In another analysis (see online analysis code and output log file), we also conduct a series of “leave-one-vessel-out” regressions using our Poisson quasimaximum likelihood estimator. The conclusions about treatment effects are robust to any single vessel being removed.

### Deviations from preregistered design

The study design or analysis deviated in four ways from the preregistered design: (i) As described above (Data collection), the field team acquired additional funding and extended the spatial and temporal scale of the original program. (ii) To better estimate the ATE_E_, the evaluation team changed the statistical estimator from a linear estimator, which is now Alternative Estimator 2 (GLS estimator), in favor of the Poisson estimator, which has been shown to be more robust in estimating mean effects from overdispersed count data like the data yielded by the RCT. (iii) The original analysis plan called for qualitative and quantitative analysis of subjective well-being based on survey data collected from a sample of households participating in the experiment, but postintervention, the evaluation team dropped that analysis because of concerns about the usefulness of the data for drawing inferences about fisher well-being. Instead, for full transparency, we report the design and quantitative results of that analysis in supplementary text 1.5 and fig. S3. Self-reported subjective well-being was higher, on average, for fishers during the trial relative to before the trial, and nearly two-thirds of fishers reported a positive or very positive opinion of the program. (iv) The original analysis plan stated that data from Tanjung Luar and Aceh Jaya would be analyzed separately. However, postrandomization, it was clear that separate analyses for each Regency suffered from low statistical power. Nevertheless, this subgroup analysis was used as part of the exploratory analysis in the main text because the live releases were concentrated in the Aceh Jaya villages.
