# Supplementary material for: Conservation impacts and hidden actions in a randomized controlled trial of a marine pay-to-release program
Source: Sci Adv. 2025 Apr 23;11(17):eadr1000. doi: 10.1126/sciadv.adr1000 (PMC13109964; doi:10.1126/sciadv.adr1000)
Supplement: Supplementary file 1 — Supplementary Text Figs. S1 to S4 Tables S1 to S4 Legend for movie S1 [file sciadv.adr1000_sm.pdf]

Science Advances
AAAS

Supplementary Materials for

Conservation impacts and hidden actions in a randomized controlled trial of a marine pay-to-release program

Hollie Booth *et al.*

Corresponding author: Hollie Booth, hollie.booth@biology.ox.ac.uk;
Thomas Pienkowski, t.pienkowski@kent.ac.uk; Paul J. Ferraro, pferraro@jhu.edu

Sci. Adv. **11**, eadr1000 (2025)
DOI: 10.1126/sciadv.adr1000

The PDF file includes:

Supplementary Text
Figs. S1 to S4
Tables S1 to S4
Legend for movie S1

Other Supplementary Material for this manuscript includes the following:

Movie S1

Supplementary Text

1.1 Robustness Checks

The results from Figure 2 and from the six robustness checks can be found in SM Tables 2-4. We also conducted an arithmetic check on the reasonableness of the estimated ATE_E from the Poisson estimator with random effects and cluster-robust standard errors (Figure 2 and Table S2, col 1). For the treated and the control conditions, we divided the raw retained catch count data for each vessel in each period by the number of days in the period, and then divided the retained catch per day per vessel in the treated condition by the same measure in the control condition (i.e., ignoring the temporal variation in period starts and stop dates and the nested structure of the data). That calculation implied a 35% increase in retained hammerhead catches and a 14% decrease in retained wedgefish catch, which are values close to those in Fig. 2.

1.2 Missing Periods

As noted in the M&M, some vessels had missing periods (no landings during a period, Figure S4). For the 26 vessels randomized to a 3-period sequence, all but 2 were observed in three periods. For the 61 vessels randomized to a 4-period sequence, 29 vessels were observed in four periods and 12 were observed in three periods. Thus 4 out of 5 vessels were observed in all or all but one of their periods, and all but 6 vessels were observed in both treated and control conditions. Among the vessels that engaged in live releases, 93% had no missing periods.

For missing periods to be a source of bias in estimating the ATE_E, missingness would have to be systematically correlated with potential retained catch outcomes in the treated or control conditions (otherwise missingness is just a source of sampling variability). We cannot observe potential outcomes, but we could look for evidence of a systematic correlation between missingness and observed treatment status.

The difference in the number of observations in the treated (127) and control (134) periods was small and not larger than we would expect by chance (one-sample test of proportion, $p=0.67$), as is the average number of periods observed in the treated (1.63) and control (1.53) conditions. Likewise, treatment status did not predict period length ($\exp(\beta) = 0.98$, $p=0.48$ (null: $\exp(\beta) =1$)).

Thus, we assumed that, in the presence of missing outcome measures for some periods, randomization of the treatment identified the average treatment effect on retained catch. Given that assumption, we used the full unbalanced panel data in our analysis: 86 vessels for a total number of 261 periods in which retained catch was observed.

1.3 Threats from interference

The internal validity of an RCT can be affected by interference ("spillovers") among the experimental units, whereby the potential outcome of one unit is affected by the treatment status of one or more other units. In the presence of interference, the results of an RCT are more challenging to interpret.

In our design, there are two types of potential interference. First, as in all RCTs in coupled-human environments, there is the possibility of interference across vessels. In our cross-over

design RCT, there is also the possibility of interference within vessels (also known as "carryover effects"). We consider the potential for both forms to exist in our study.

1. Within-vessel, across-periods interference, whereby a vessel's treatment condition in one period affects its retained catch in the control condition, or vice-versa. By estimating the treatment effect only during the first period, we address, in the main text, the threat that the effects of treatment exposure in one period could carry over into subsequent treatment or control conditions. We cannot further discern across-period interference by estimating the treatment effect separately by period and then comparing effects in the earlier periods to the later periods because we lack sufficient variation in the sequence of treatment assignment across vessels. Without more variation in the sequence of assignment, we cannot differentiate carry-over effects from the moderating effects of seasonality.

Another form of within-vessel, across-period interference are "anticipatory effects," where a vessel's expectation of future treatment exposure affects their behavior in the control condition. With our data and design, we cannot rule out this form of within-vessel interference, but this form seems unlikely in our study context. The most plausible mechanism for such an effect would require a well-functioning credit market, whereby control vessel owners could borrow funds against their expected future release payments and then invest those funds in a way that would affect retained catch of the two target species. Such a mechanism would require well-functioning credit markets, which do not exist, and a clear expectation by the borrower and lender about the number of endangered species that would be released, verified, and compensated in the treated condition. Credit markets function poorly in our study area (limited credit, high interest rates) and given that the pay-to-release program was new, borrowers and lenders would not know the likely payment levels.

2. Across-vessel interference, whereby a vessel's retained catch in treated or control conditions are affected by other vessels' treatment status. This interference is most likely to arise within-period, but it could also arise across-periods. The three major mechanisms for this form of interference in our study context are:

a. *Market-mediated price effects* that could occur because treatment induces a change in the supply of certain species, which subsequently affects prices for all species. Were this interference to exist, it would likely exist within villages. The five villages are too small a part of the market and too spread out to affect to affect prices across villages. Interference from market-mediated price effects requires landings markets to be competitive, so that prices would be very responsive to modest changes in supply of the two target species that could arise from the pay-to-release program (and for vessels to be responsive to those changes in prices). Buyers, however, operate at the district level (i.e., a village has only one buyer, on average) and information about supply moves slowly. Thus, these markets are not well characterized as competitive markets.

Nevertheless, we consider what such interference would look like in our study context, and we develop a statistical approach that, under certain assumptions, eliminates its effects from the estimation procedure. If the treatment were to lead to an increase in retained catch in species X (as we believe it did for hammerheads), it could lower the price that both treated and control

vessels receive, which would lower the control vessels retained catch of species X and, perhaps, change the retained catch of species Y (the direction is not certain but rather depends on whether species X and Y are complements in production or substitutes). In this case, the interference will make the treatment look less effective than it was for species X (control boats retained fewer fish than they otherwise would have) and it would have an ambiguous effect on the estimated treatment effect for species Y. But the only way that a pay-to-release program can increase retained catch of any fish is for there to be hidden action. Thus, the threat from this form of interference is that we would over-estimate the magnitude of the hidden action, not spuriously claim that it exists. If, instead, the treatment were to lead to a decrease in retained catch in species X (as we believe it did for wedgefish), it could increase the price that both treated and control receive, which would increase the control groups retained catch of species X and, perhaps, change the retained catch of species Y (the direction is not certain but rather depends on whether species X and Y are complements in production or substitutes). In this case, the interference will make the treatment look more effective than it is for species X (control boats retained more fish than they otherwise would have retained) and have an ambiguous effect on the treatment effect of species Y. Thus, the threat from this form of interference is that we would under-estimate the magnitude of the hidden action for species X.

Alternatively, we can consider an estimation approach that would be robust to market-mediated interference, under certain assumptions about the nature of the interference. Recall that, in each period, roughly 50% of the sample is randomly assigned to the treatment condition. Let us assume that when 50% of the sample is treated in a period, market-mediated interference exists in that period (i.e., the treatment induces a change in prices for the target fish in that period). This interference would complicate the interpretation of any estimator that leverages the within-period (cross-sectional) variation in treatment status across vessels, as our main estimator does. Yet, an estimator that only uses the *within-vessel* variation will not suffer the same problem if we also assume no carryover effects (no within-vessel interference) and we assume the price-mediated interference from treating 50% of the sample is constant across periods. In other words, if we can make the plausible assumption that any price effects from the treatment are the same in each period (i.e., we assume common responses, on average, to common price shocks across periods), we could use a two-way, fixed-effects estimator that leverages only the within-vessel variation in treatment across periods and uses period-by-village dummy variables to control for the effects of different time periods. Although this estimator may have the same external validity issue that we already have (would the estimated treatment effects be the same if fish prices were different?), it would not suffer from threats to internal validity because, within-vessel, the price effects of treating ~50% of the sample are the same in the vessel's treated and control conditions. When we use only within-vessel variation, we lose statistical power (precision) but we remove the effect of any price-mediated across-vessel interference. If we obtain roughly the same estimated effect as in our main estimator, that pattern would imply no substantial market-mediated interference. The estimated effect. For both target species, the estimated effect is nearly identical: for hammerheads, we estimate an increase in retained catch of 38% [95% CI: 3% - 84%], and for wedgefish, we estimate a decrease in retained catch of 27% [95% CI: -50% - 8%].

Note that this alternative estimator not only addresses one form of interference, it also addresses any concerns that, despite the randomization of the treatment assignment sequences, the estimated effects (i.e., the conclusions) could be a result of severe imbalance in unobservable pre-experiment predictors of post-experiment retained catch. The estimator addresses those concerns because the estimator only uses the within-vessel variation in the treatment condition, thereby controlling for unobserved, time-invariant differences across the randomized sequences that arose by chance.

b. *Stock-mediated effects* that could occur because treatment induces a change in how many fish are caught by some vessels, which affects the stock available to other vessels and thus their retained catch. With our data and design, we cannot rule out this form of across-vessel interference, but this form seems unlikely in our study context. The vessels are small and the fishery is large, and thus individual boats are unlikely to have a detectable effect on stock in the short time horizon of the RCT. If one were willing to assume that there are no within-vessel forms of interference and that any stock-mediated effects are a function of the percent of vessels treated, and thus are constant across time periods because the percent of vessels treated is roughly constant, then the alternative estimator used to eliminate the effect of price-mediated interference would also do the same for stock-mediated effects.

c. *Location-mediated* effects that could occur because treatment induces a change in where vessels fish, which subsequently affects the retained catch of other vessels via displacement of vessels to other areas or via subsequent stock-mediated effects. This form of interference could happen within-villages or across-villages but would be most likely within-villages because fishing areas are village-specific. With our data and design, we cannot rule out this form of across-vessel interference, but this form seems unlikely in our study context for the same reason that stock-mediated effects seem unlikely.

3. *A combined form of within-vessel and across-vessel interference* that could occur if vessels colluded among themselves to extract more payments from the pay-to-release program. By “collusion,” we mean vessels coordinate their fishing behaviour to maximize their collective payments from the pay-to-release program and then share among themselves the incremental gains that were garnered through the collusion. For vessels to collude, vessels in the control condition would, contrary to their original plans, avoid the best locations for catching the target species and vessels from the treated condition would purposely go there. If we assume that vessels are rational, which would likely be a required assumption for them to engage in collusion of this degree of sophistication, then they would have chosen the individually optimal locations in the absence of collusion. Thus, ignoring the release payments, both groups will end up, on average, with lower profits in the collusion case than they otherwise would have had. If that were not the case, then the vessels would not need to have altered their behaviors under the collusive agreement. For this collusive outcome to be optimal (i.e., to induce participation): (i) the fishers must expect that the risk-adjusted present value of the additional payments garnered via the collusion would be greater than the risk-adjusted present value of lost profits from changing their no-payment fishing plans (risk-adjusted because fishers care about uncertainty and there’s a risk that the extra release payments will not cover the lost profits); and (ii) the control vessels must expect that the treated vessels would, at the end of the period, share their publicly unobservable

spoils from collusion with the control vessels ("publicly unobservable" because how much treated vessels are paid by the pay-to-release program is only observable to the recipient) or, implausibly, the control vessels would have to assume that the spoils will be equal in each period and thus each group gets its due when it is in the treated state (implausible both because some villages only do three periods and, even in the four-period villages, fishers know that catch is stochastic across time). Such collusion would require a lot of trust and novel coordination among vessels and, given the relatively small release payments, we believe it is unlikely that fishers would incur the substantial costs and risks to organize themselves to try to increase their release payments beyond which they would achieve in the absence of collusion. Moreover, in none of our surveys and interviews, as well as in none of the many informal conversations among fishers and field staff throughout the RCT, did our field team observe reports of any behavior that would be consistent with collusion.

1.4 Exploratory Analyses

As discussed in the Introduction in the main text, there are four potential countervailing behavioural mechanisms (pathways) that could make a conservation incentive program not as effective as anticipated: (1) Hidden information; (2) Hidden action; (3) Relaxation of budget constraints; and (4) Multiple Margins or Spillovers. In the pay-to-release RCT, hidden information was not a potential countervailing mechanism because we can assume, based on field knowledge, that no vessels released live fish in the absence of payments because the retained catch had market or consumptive values. The other three mechanisms are consistent with $ATE_C < ATE_E$, and, if not all caught fish were released alive, the mechanisms also could have led to $ATE_E > 0$ (overall mortality increases, on average).

Spillovers, whereby changes in how one species is fished affect the retained catch of the other species, can arise in two ways. First, actions to release species A alive may lead to more retained catch of species B, or vice-versa (i.e., a spillover that is associated with the conservation incentive working in the desired way for one or both species, but the technique used to release one species alive affects the retained catch of the other species). Second, actions to increase the catch of species A may lead to more retained catch of species B, or vice-versa (i.e., a spillover that is associated with hidden action for one or both species: efforts to catch more of species A not only increase the retained catch of species A but also increase the retained catch of species B). Based on field knowledge, we eliminated the first version of spillovers in our context – there was nothing about the procedure of releasing one species alive that would have increased the retained catch of another species. Thus, in our study context, the spillover (multiple margins) issue arose only in the presence of hidden action.

To assess the potential for spillovers, we conducted an exploratory analysis of the subgroup of villages that accounted for most of the live releases (acknowledging that our statistical power declined in this subgroup analysis). The three villages from the Aceh Jaya regency accounted for 99% of the live releases. In these villages, the period-level correlation of retained catches of hammerhead and wedgefish within each vessel in the control condition was large and positive (ρ=0.38). Thus, spillovers were possible in the treated condition if hidden action induced vessels in these villages to catch one or both species. In the other villages, this correlation was near zero (ρ=0.07). Moreover, this retained catch correlation increased in the treated condition for the three villages receiving the most payments (ρ=0.53) but not in the other villages (ρ=0.03).

To further explore the potential for spillovers, we focused on the two villages that received 98% (467/475) of the live releases of wedgefish. These two villages released few hammerheads in the experiment (23/364), and one vessel accounted for 78% of those releases. Thus, if there was only hidden action, the live release payments would have increased retained wedgefish catch, but not retained hammerhead catch, in the treated condition compared to the control condition (i.e., ATE_C (wedgefish)> ATE_E (wedgefish)). Given that the live hammerhead releases were few, we would expect no difference between the retained catch in treated and control conditions in the absence of spillovers; i.e., ATE_E (hammerheads) = 0. However, if there were spillovers from the increase in wedgefish catch, $ATE_E > 0$ for retained hammerhead catch.

Using the Poisson estimator from the main analysis for this subgroup analysis, we observed evidence of both hidden action for the wedgefish catch and spillovers to the hammerhead catch. The offer of payments reduced wedgefish retained catch by an estimated ATE_E (wedgefish) = -37% [95% CI: -61%, 2%], which is smaller ($p<0.01$) than the estimate from the conventional approach (ATE_C (wedgefish) = -80%).[1] The offer of payments also increased retained hammerhead catch by an estimated ATE_E (hammerhead) = 59% [95% CI: 3%, 144%], which is statistically different from zero ($p<0.05$). Thus, these results suggested that at least some of the countervailing effects observed in the RCT arose from a mix of hidden action related to the wedgefish payments and a subsequent spillover to the hammerhead catch.

We conducted a similar analysis in the one village that released 94% of the hammerheads and only released 6 wedgefish from one vessel. Although this subgroup was small, and thus the estimated effects were imprecise, we observed patterns consistent with hidden action and spillovers. The offer of payments increased hammerhead retained catch by an estimated ATE_E (hammerhead) = 46% [95% CI: -12%, 143%], which was a different size and magnitude ($p<0.05$) than the estimate from the conventional approach (ATE_C (hammerhead) = 16%).[2] The offer of payments also increased wedgefish retained catch by an estimated ATE_E (wedgefish) = 48% [95% CI: -41%, 269%], which was large and positive but imprecisely estimated (not statistically different from zero). The point estimates imply that at least some of the countervailing effect observed in the RCT arose from a mix of hidden action related to the hammerhead payments and a subsequent spillover to the wedgefish catch, but there was much more noise in this estimation than for the villages in the previous paragraph.

Field evidence and exploratory statistical analysis imply that the budget-relaxation mediation pathway was not an important countervailing pathway. For live releases in one week, recipients were paid in the subsequent week. Thus, some vessels were paid money during their first treated period, and a budget-relaxation pathway could have been activated. Yet, field knowledge also implies that fishing investments take time to complete, and their effects were likely to be cumulative (perhaps nonlinearly). Thus, if budget-relaxation was an important pathway, we

---

[1] Vessels in this subgroup released 467 wedgefish and retained 116 wedgefish in the treated periods, thus the conventional approach to estimating the ATE based on live releases only would imply that the offer of payments reduced retained wedgefish by 467/(116+467)= 80%.

[2] Vessels in this subgroup released 341 hammerheads and retained 1744 hammerheads in the treated periods, thus the conventional approach to estimating the ATE based on live releases only would imply that the offer of payments reduced retained hammerheads by 341/(1744+341) = 16%

would expect the ATE_E for both species to be larger in the third and fourth periods compared to the first and second periods (i.e., larger in the second round of treatment). Although we acknowledge that our design had weaker statistical power for this subgroup analysis than the full sample analysis, we did not detect this pattern in our data. The estimated ATE_E was larger in the earlier periods (not statistically significant), which was the opposite of the expected pattern if budget-relaxation were an important driver.

1.5 Subjective well-being and qualitative narratives

The field team collected data on fishers' subjective well-being, perceptions of the program and reasons for participation or non-participation using interview-administered surveys, before and during the intervention from vessels in the treatment and control conditions. The team also conducted end-of-trial semi-structured interviews with female heads of household. Survey instruments are available at: https://osf.io/b27ja/?view_only=39fe7b0d223547a8a2cb92e217cdea99.

The team used five-point Likert-scaled questions to collect quantitative scores (very bad (-2) to very good (+2)) on a) subjective well-being and b) perceptions of the program, followed by open-ended questions for respondents to explain their answers. The average reported subjective well-being was higher for treatment than for control fishers in the first period (Figure S2). Almost all interviewed fishers and female heads of household reported positive perceptions (60%, N = 62; 100%, N = 13, respectively) or neutral perceptions (39%, N = 41) of the program. Only one respondent reported a negative perception of the program. Reasons for positive perceptions of the program broadly related to "helping fishers' livelihoods and protecting fish" (e.g., "*…Karena sangat membantu mata pencaharian nelayan dan ikut serta dalam menjaga pelestarian ikan*"). Fishers who did not report any releases but still had positive perceptions of the program reported that they appreciated that they were "not feeling pressured" by the program (e.g., "*tidak terlalu ditekan dalam proses uji coba kompensasi ini*").

The team also asked fishers if they had submitted a live release video and received a compensatory payment, which was followed by open-ended questions to explain (if yes) their reasons for participating and what the payments had been spent on or (if no) reasons for not participating. Fishers who received release payments stated that they participated because it helps fishers while supporting shark conservation, with "no pressure" to participate (e.g., "*Karena program sangat membantu nelayan dan tidak ada tekanan sama sekali, dan populasi hiu bisa tetap berkembang*" and/or because they were following their friends (e.g., "*Karena banyak teman-teman yang ikut berpartisipasi*"). They reported that payments helped to cover their family's daily needs, such as food, school fees and fishing operating costs (e.g., fuel, fixing nets). Moreover, when asked to explain why they participated and why they had positive perceptions of the program, some fishers made statements that were consistent with the experimental results that imply that payments could have induced increased fishing effort, such as "*Aku ikut karena harga kompensasi lebih mahal dari harga pasar*" (I participate because the price of the compensation is higher than the market price); "*Proyeknya sangat membantu nelayan, serta kompensasi yang diberi lumayan mahal*" (the project really helps fishers because the compensation given is pretty expensive); "*Aku senang dengan proyeknya karena ada keuntungan lebih dibandingkan di jual di pasar*" (I am happy with the program because there are more profits than selling at the market). The primary reasons given for not participating were that

they had not yet caught the target taxa or had not yet found an individual still alive (e.g., "*Belum mendapatkan hiu yang dimaksud"* (have not yet caught a shark that is included [in the program]).

Fig. S1.

The distribution of total retained catch of wedgefish (WF, a.) and hammer head (HH, b.) sharks among the 261 vessel-period observations.

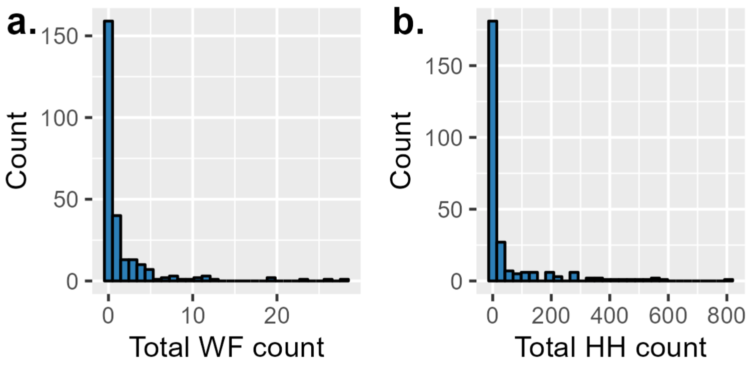

Fig. S2.

An illustrative diagram of the data aggregation process. Retained catch and live releases were aggregated for each vessel in each phase, yielding the total retained catch and live release for a given phase for each vessel, as illustrated with archetypical synesthetic data below.

Vessel	Phase	Landing	Release
Boat_1	1	5	1
Boat_1	1	10	5
Boat_1	2	20	10
Boat_1	2	10	5
Boat_1	2	0	0
Boat_2	1	5	5

→

Vessel	Phase	Vessel-phase	Landing	Release
Boat_1	1	Boat_1_1	15	6
Boat_1	2	Boat_1_2	30	15
Boat_2	1	Boat_2_1	5	5

Fig. S3.

Subjective well-being – from very bad (-2) to very good (+2) – for treatment and control fishers during the first rotation of the RCT, where (A) is a bar plot of Likert categories and (B) is a violin plot of numerical Likert scores.

A

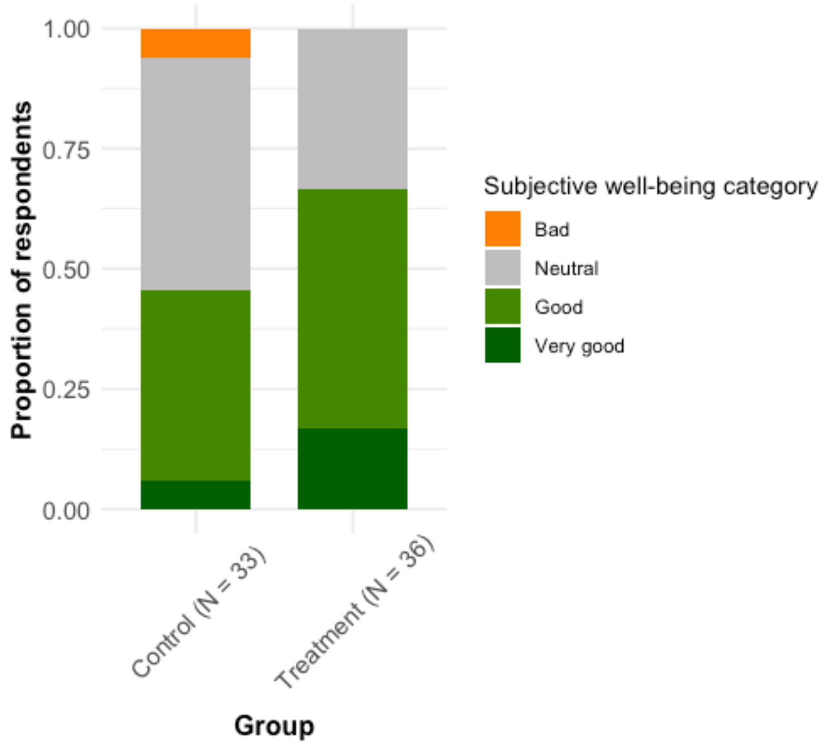

B

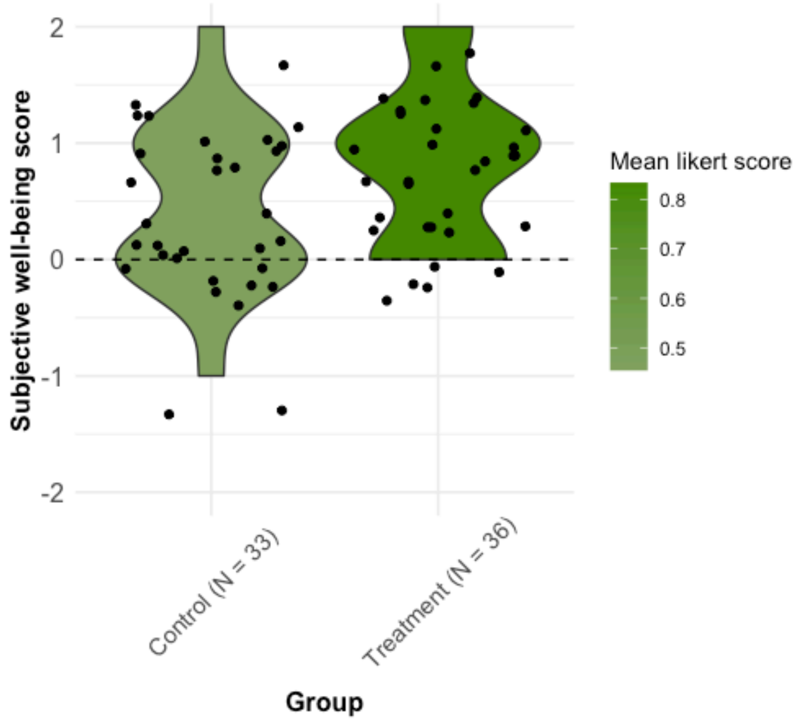

Fig. S4.

Patterns of Observed Periods (1) & Unobserved Periods (.)

3-Period Vessels

Freq.	Pattern
23	111
3	11.

N=26

4-Period Vessels

Freq.	Pattern
29	1111
9	111.
7	.11.
3	.111
3	11..
2	.1.1
2	1...
1	...1
1	..1.
1	..11
1	.1..
1	11.1
1	….

N=61

Table S1.

Intervention cost reporting worksheet. US$ 1 ~ IDR 15,000

Category	Description	Unit Cost	Currency	Units	Fixed/ Variable	Total cost (IDR)	Total cost (USD)	Notes
Labour	Project coordinator	4,500,000	IDR	14	Variable	63,000,000	4,200	Monthly stipend
Labour	Field Assistants	3,600,000	IDR	14	Variable	50,400,000	3,360	Monthly stipend
Consumable	Waterproof cameras	1,500,000	IDR	45	Variable	67,500,000	4,500	Cameras to record releases
Consumable	Project merchandise	500,000	IDR	87	Variable	43,500,000	2,900	T-shirts and bags
Consumable	Payments to fishers - wedgefish Aceh Jaya	120,000	IDR	473	Variable	56,760,000	3,784	
Consumable	Payments to fishers - wedgefish East Lombok	2,000,000	IDR	2	Variable	4,000,000	267	
Consumable	Payments to fishers - hammerheads Aceh Jaya small	15,000	IDR	268	Variable	4,020,000	268	
Consumable	Payments to fishers - hammerheads Aceh Jaya large	50,000	IDR	96	Variable	4,800,000	320	
Consumable	Workshops and training materials	5,000,000	IDR	2	Variable	10,000,000	667	Engagement workshop, contract printing, release guidelines
**Grand total**						**303,980,000**	**20,265.33**	

Table S2.

Estimated Treatment Effects for Hammerhead Sharks.

	(1) Original	(2) Period 1	(3) Recipients	(4) Multi-level	(5) Bootstrapped SE	(6) Outliers
Offer Payment	1.437	7.866	1.501	1.439	1.437	1.452
	[1.076,1.919]	[3.308,18.70]	[1.073,2.100]	[1.075,1.926]	[1.031,2.004]	[1.090,1.936]
Conventional	0.956	0.999	0.945	0.956	0.956	0.956
N	261	70	92	261	261	258

NOTE: Estimated effects of offering live release payments on retained catch. Estimates are presented as Incidence Rate Ratios (IRR) ([IRR-1]*100 yields the percent change). In brackets are the estimates' 95% confidence intervals, based on cluster-robust standard error estimates. "Conventional" refers to the estimated effect from the conventional monitoring and evaluation in the IRR format, and N refers to the sample size. (1) Original Poisson estimator (described in M&M; results in Figure 2); (2) Period 1 – Original estimator using only Period 1 data; (3) Recipients - Original estimator using only payment recipient data; (4) Multi-level – Alternative Poisson estimator using multi-level, mixed-effects model; (5) Bootstrapped SE - Original estimator with cluster-robust bootstrapped errors; and (6) Outliers - - Original estimator after removing top 1% of outcome variable values.

Table S3.

Estimated Treatment Effects for Wedgefish.

	(1) Poisson	(2) Period 1	(3) Recipients	(4) Multi-level	(5) Bootstrapped SE	(6) Outliers
Offer Payment	0.752	1.540	0.732	0.742	0.752	0.786
	[0.513,1.103]	[0.470,5.043]	[0.445,1.204]	[0.506,1.087]	[0.474,1.194]	[0.565,1.093]
Conventional	0.294	0.332	0.231	0.294	0.294	0.294
N	261	70	92	261	261	258

NOTE: Estimated effects of offering live release payments on retained catch. Estimates are presented as Incidence Rate Ratios (IRR) ([IRR-1]*100 yields the percent change). In brackets are the estimates' 95% confidence intervals, based on cluster-robust standard error estimates. "Conventional" refers to the estimated effect from the conventional monitoring and evaluation in the IRR format, and N refers to the sample size. (1) Original Poisson estimator (described in M&M; results in Figure 2); (2) Period 1 – Original estimator using only Period 1 data; (3) Recipients - Original estimator using only payment recipient data; (4) Multi-level – Alternative Poisson estimator using multi-level, mixed-effects model; (5) Bootstrapped SE - Original estimator with cluster-robust bootstrapped errors; and (6) Outliers - - Original estimator after removing top 1% of outcome variable values.

Table S4.

Estimated Treatment Effects for Hammerheads and Wedgefish

	(1) Hammerheads	(2) Hammerheads/Day	(3) Wedgefish	(4) Wedgefish/Day
Offer Payment	10.02	0.0980	-0.311	-0.0018
	[-1.247,21.29]	[-0.0231,0.219]	[-1.013,0.392]	[-0.0074,0.0038]
Percent Change	0.240	0.260	-0.170	-0.120
N	261	261	261	261

-

NOTE: Estimated effects of offering live release payments on retained catch, based on a Generalized Least Squares, random-effects panel data estimator. Estimates are presented as counts of fish. In brackets are the estimates' 95% confidence intervals, based on cluster-robust standard error estimates. Percent Change refers to the percent change in retained catch implied by the estimated effect (0.24 means 24%) and N refers to the sample size. Recall that the conventional monitoring and evaluation estimated percent changes were a 4% (-0.04) reduction for hammerhead retained catch and 71% (-0.71) for wedgefish retained catch

Movie S1.

A selection of live release videos submitted by fishers in the pay-to-release program.
